# REDUCE PORT LAPAROSCOPIC SPLENECTOMY FOR GIANT EPITELIAL CYST

**DOI:** 10.1590/S0102-6720201500040016

**Published:** 2015

**Authors:** Mariano PALERMO, Luis BLANCO, Pablo ACQUAFRESCA, Jose MENENDEZ, Rafael GARCIA

**Affiliations:** From the Department of Surgery, Hospital Nacional Prof. A. Posadas, University of Buenos Aires, Buenos Aires, Argentina.

**Keywords:** Laparoscopy, Splenectomy, Surgery

## Abstract

**Background:**

Delaitre and Maignien performed the first successful laparoscopic splenectomy in 1991. After that, laparoscopic splenectomy has become one of the most frequently performed laparoscopic solid organ procedures.

**Aim:**

To demonstrate the surgical techique of laparoscopic splenetomy with reduced portals.

**Methods:**

A reduce port laparoscopic splenectomy was performed by using a 10 mm and two 5 mm trocars. To entered the abdomen a trans-umbilical open technique was done and a 10 mm trocar was placed. A subcostal 5 mm trocar was placed under direct vision at the level of the anterior axillary line and another 5 mm port was inserted at the mid-epigastric region. Once it was completely dissected and freed from all of its attachments the hilum, splenic artery and vein, was clipped with hem-o-lock and divided with scissors. Then an endobag was used to retrieve the spleen after being morcellated trough the umbilical incision.

**Results:**

This technique was used in a 15 years old female with epigastric and left upper quadrant pain. An abdominal ultrasound demonstrated a giant cyst located in the spleen. Laboratory tests findings were normal. The CT scan was also done, and showed a giant cyst, which squeeze the stomach. The patient tolerated well the procedure, with an unremarkable postoperative. She was discharge home 72 h after the surgery.

**Conclusion:**

The use of reduce port minimizes abdominal trauma and has the hypothetical advantages of shorter postoperative stay, greater pain control, and better cosmesis. Laparoscopic splenectomy for giant cysts by using reduce port trocars is safe and feasible and less invasive.

## Abstract (pt)

**Racional:**

Delaitre e Maignien realizaram a primeira esplenectomia laparoscópica bem sucedida em 1991. Depois disso, a esplenectomia laparoscópica tornou-se um dos procedimentos laparoscópicos de órgãos sólidos mais frequentemente realizadas.

**Objetivo:**

Demonstrar a técnica cirúrgica de esplenectomia laparoscópica com portais reduzidos.

**Métodos:**

A esplenectomia laparoscópica reduzida em potais foi realizada usando um trocarte de 10 mm e dois de 5 mm. A entrada abdominal se dá pela técnica aberta trans-umbilical e um trocarte de 10 mm foi aí colocado. Um de 5 mm subcostal foi colocado sob visão direta no nível da linha axilar anterior e outra trocarte de 5 mm foi inserido na região médio-epigástrica. Uma vez completamente dissecado e liberado todos os seus ligamentos do hilo, a artéria e veia esplênicas foram ligadas com hem-o-lock e seccionado com tesoura. Em seguida, um Endobag foi usado para retirar o baço após ser ele morcelado através da incisão umbilical.

**Resultado:**

Esta técnica foi utilizada em uma jovem de 15 anos com dor epigástrica e em hipocôndrio esquerdo. Ultrassom abdominal mostrou um cisto gigante localizado no baço. Exames complementares tiveram resultados normais. TC mostrou um cisto gigante, que projetava-se para o estômago. A paciente tolerou bem o procedimento, com pós-operatório normal. Ela teve alta hospitalar em 72 h após a operação.

**Conclusão:**

A diminuição de portais minimiza o trauma abdominal e tem as vantagens conhecidas de menor tempo de pós-operatório, maior controle da dor, e melhor efeito cosmético. A esplenectomia laparoscópica para cistos gigantes usando trocárteres reduzidos é segura, viável e menos invasiva.

## INTRODUCTION

Splenectomy was initially described for hereditary spherocytosis by Sutherland and Burghard in 1910 and for idiopathic thrombocytopenic purpura by Kaznelson in 1916^8^. It has been well recognized as an effective cure for hematologic disorders, better than medical treatment. The first successful laparoscopic splenectomy was performed by Delaitre and Maignien in 1991^7^^,^ 8. After that, laparoscopic splenectomy has become one of the most frequently performed laparoscopic solid organ procedures.

Laparoscopic splenectomy is emerging as the gold standard for the management of various hematologic disorders. Minimally invasive surgery has earned boundless acceptance. The enthusiasm to limit the trauma of large incisions has been the incentive of the development of minimally invasive surgery during the past century. Numerous technology and equipment in laparoscopy have been emerging. It started gradually reducing and repositioning trocars during laparoscopic surgery. It began with repositioning the subxiphoid trocar into the umbilicus. Less trocars equals less abdominal trauma. In small spleens a single port laparoscopic surgery can be performed. In large spleens, less trocars can be used. 

The aim of this artcile is to details the splenectomy procedure using only three trochars.

## METHOD

### Surgical technique

The patient is placed in lateral decubitus, to enter the abdomen using a trans-umbilical open technique and a 12 mm trocar is placed. Through it a 10 mm 30^o^ scope is inserted. A subcostal 5 mm trocar is placed under direct vision at the level of the anterior axillary line and another 5 mm port is inserted at the midepigastric region. Using a 5 mm harmonic scalpel (Harmonic Ace, Ethicon) and 5 mm instruments, access is gained to the lesser sac by dividing the gastrosplenic ligament and short vessels until the upper pole of the spleen. The splenic flexure of the colon is mobilized to get the lower pole of the spleen freed. The posterior splenorenal ligament is then freed. 

Once the spleen is completely dissected free from all of its hilum attachments, splenic artery and vein are clipped with hemolocks and divided with scissors. It is suggested clipping the artery first and then the vein, this can reduce the size of the spleen in an important percentage. This is specially useful when dealing with great size spleen. Then an endobag is used to retrieve the spleen after being morcellated through the umbilical incision. A drain, exteriorized through the lateral 5 mm trocar is used routinely. 

## RESULT

**FIGURE 1:**
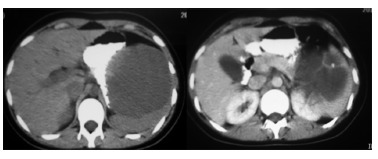
- CT scan showed a giant cyst which squeeze the stomach

This technique was used in a 15 years old female refered due to epigastric and left upper quadrant pain. An abdominal ultrasound was performed in order to find gallblader stones, but a giant cyst located in the spleen was found. Laboratory tests were normal. The CT scan showed a giant cyst which squeeze the stomach (Figure 1). The patient was scheduled for surgery, and a laparoscopic approach was performed.

**FIGURE 2:**
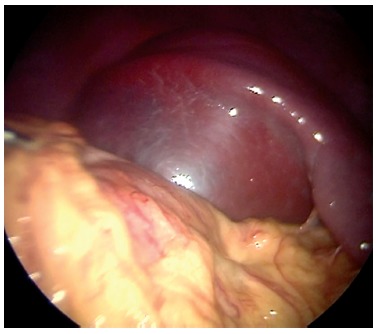
- Gian cyst located in the spleen is observed when entering the abdomen

**FIGURE 3:**
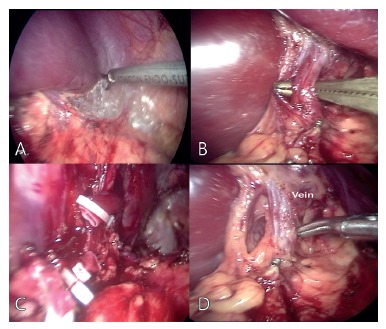
- Technical steps: A) ligaments were divided with electronic shears; B, C and D) splenic vein and artery being ligated by using hemolocks

Lateral positioning was used and with open technique the abdomen was entered. A 10 mm trocar was inserted at the umbilicus and two more trocars were placed in the left upper quadrant. Once the abdomen was entered a gian cyst located in the spleen was observed (Figure 2). The ligaments were divided with electronic shears (Figure 3 A) and the splenic vein and artery were ligated by using hemolocks (Figure 3 B-D)

**FIGURE 4:**
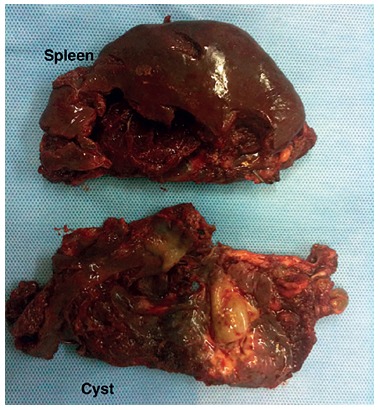
- Spleen and cyst are observed after resection

Finally the spleen was placed into the retrieval bag and removed from the abdominal cavity before morcellating it with ringed forceps. In the Figure 4, the spleen and cyst are observed after its resection. 

**FIGURE 5:**
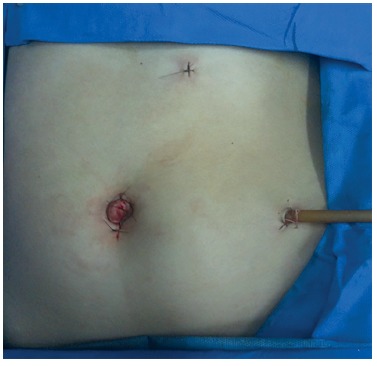
- Final aspect of the operation, and a drain exteriorized through the lateral 5 mm trocar is used routinely

The patient tolerated well the procedure, with an unremarkable postoperative period. She was discharge home 72 h after the surgery with all the vaccines set (Figure 5).

## DISCUSSION

The first successful laparoscopic splenectomy was performed by Delaitre and Maignien in 1991^7^^,^ 8. After that, laparoscopic splenectomy has become one of the most frequently performed laparoscopic solid organ procedures.

Regarding the surgical technique, the lateral positioning is the preferred approach for laparoscopic splenectomy because the gravity effect pulls down the colon, stomach and omentum and allows better visualization of the spleen. Three left subcostal ports are usually adequate for normal-sized spleens. In the presence of splenomegaly, ports are optimally positioned 4 cm below the inferior tip of the spleen, parallel to the left costal margin, but within reach of the diaphragm. If the spleen is extremely large, the trocars may have to be placed substantially more inferiorly than normal, creating the need for an additional port posteriorly. This port allows for lateral retraction of the spleen and can facilitate access to the diaphragmatic attachments. Additional ports are placed under laparoscopic guidance. Visualization and efficiency are optimized by exchanging the camera between the medial and lateral ports, while the surgeon operates with both hands^8^.

Supermassive spleens are most effectively managed with a hand-assisted approach. This approach utilizes similar patient positioning but uses a hand-assisted device to facilitate insertion of the surgeon's nondominant hand into the abdominal cavity while maintaining pneumoperitoneum. This technique allows for improved tissue handling and atraumatic manipulation of the enlarged spleen. For patients with supermassive spleens, lateral positioning is altered slightly. In these cases, the patient is placed supine with the left side elevated at 45°. This allows the surgeon to take advantage of gravity while also providing comfortable access through the hand-assist incision.

Depending upon the hand dominance of the surgeon, the hand-assist device can be placed in either a midline (right-hand dominant) or a subcostal position (left-hand dominant) through a 7 to 8 cm incision which is located 2 to 4 cm caudal to the inferior pole of the enlarged spleen. In both situations, the surgeon stands on the right side of the patient. The nondominant hand is inserted through the hand-assist device and provides medial retraction and rotation of the spleen. The hilar pedicle is transected with an endoscopic gastrointestinal anastomotic stapler utilizing a vascular cartridge. The spleen is placed into an appropriately-sized impermeable retrieval bag. This bag must be strong enough to avoid rupture during morcellation and extraction of the specimen. Placing the spleen into the retrieval bag can be one of the most time-consuming and challenging aspects of the operation but it's necessary in order to avoid possible splenosis which is unwanted in many hematologic disease. The patient is placed into Trendelenburg's position and the spleen is gradually directed into the bag. After the spleen is within the retrieval bag, the opening of the bag is delivered through the largest port site or the hand-assist incision and the spleen is morcellated with ringed forceps.

The most common hematologic disorder that requires splenectomy is the idiopathic thrombocytopenic purpura where surgery is indicated in patients with refractory symptomatic thrombocytopenia after 4 to 6 weeks of medical therapy, patients requiring toxic doses of steroids to achieve remission, and patients who relapse following an initial response to steroid therapy. Hereditary spherocytosis (hereditary hemolytic anemia), is curable in approximately 90% of patients after splenectomy. Surgery is indicated for all patients with hereditary spherocytosis and splenomegaly, for patients with symptoms of severe hemolytic anemia or mild hemolytic anemia and concomitant gallstones, and for patients with cholelithiasis in siblings^8^.

Other indications for splenectomy are HIV-related, thrombocytopenic purpura systemic, lupus erythematosus-related, thrombocytopenic purpura, thrombotic thrombocytopenic purpura, autoimmune hemolytic anemias, Hodgkin's disease, non-Hodgkin's lymphoma, chronic lymphocytic leukemia and Hairy cell leukemia^8^.

The contraindications to laparoscopic splenectomy can be divided into: absolute contraindications like, severe cardiopulmonary disease or cirrhosis with portal hypertension. And relative contraindication are previous abdominal surgery (open Hasson technique is mandatory) and some authors include massive splenectomy^1^.

Laparoscopic splenectomy is emerging as the gold standard for the management of various hematologic disorders. Since the first laparoscopic splenectomies were performed in adults (1991) and children (1993), laparoscopic splenectomy has become the gold standard for the elective removal of normal sized spleens. It clearly has been shown to have less morbidity and postoperative pain, shorter hospital stay, earlier return of bowel function, and superior cosmesis compared with open splenectomy^5^^,^ 6. 

Although laparoscopic splenectomy is superior to open splenectomy for normal sized spleens, splenomegaly has been considered a contraindication to the laparoscopic approach because of difficulties with bleeding and removal from the abdomen. Over the last years, however, several authors have reported laparoscopic removal of enlarged spleens. With the development of hand-assisted laparoscopic surgery, the retraction of massive spleens was technically feasible. Recent studies, have shown that, aside from operative time, there is no difference in transfusions, length of stay, morbidity, or conversion rate with laparoscopic splenectomy for large spleens compared with normal sized spleens.

Laparoscopic techniques, surgical skills, and instrumentation have improved, so are safety and efficacy even in the presence of splenomegaly.

Authors like Kent W. Kercher et al.^8^, made a study on 177 patients who underwent laparoscopic splenectomy where forty-nine patients (28%) were identified as having massive splenomegaly. They defined massive splenomegaly as a craniocaudal length ≥17 cm or a weight ≥600 g. Spleens greater than 22 cm in craniocaudal length, 19 cm in width, or a weight greater than 1600 g were defined as "supermassive." And in children, splenic size greater than four times normal for age was defined as massive. They recommend that most surgeons acquire their initial laparoscopic experience with normal-sized spleens prior to attempting laparoscopic splenectomy of splenomegaly. And the author conclude that laparoscopic splenectomy has become the gold standard for elective splenectomy in patients with normal-sized spleens and laparoscopic splenectomy in the setting of massive splenomegaly is safe and effective providing distinct advantages over the open operation. In the presence of supermassive splenomegaly, the use of hand-assisted laparoscopic surgery maintains the benefits of a minimally invasive approach^8^.

Other authors like Arin K. Greene ^7^, have used the Lahey bag to remove a massive spleen. The author describe that this technique facilitate the removal of massively enlarged spleens laparoscopically (>1,000 g), because a large abdominal incision to remove the spleen is not required. The spleen is broken up while in the Lahey bag so the risk of splenosis is eliminated.

Because of suitable single port laparoscopy equipment is not available to may centers due to cost, Colon et al.^3^ developed a technique where additional instrumentation was kept to a minimum. Boone et al.^2^ demonstrate in their series that single port splenectomy is safe and feasible in an unselected patient population, their patient population included patients with prior surgery, obese patients, medical comorbidities, splenomegaly, and severe thrombocytopenia. In their study they also compared it to standard laparoscopic splenectomy. The results showed no statistically significance in morbidity and mortality between both groups. Analysis of postoperative pain medication requirement revealed that the single incision patients required fewer narcotics, but this did not reach statistical significance either. Single port splenectomy was associates with a significantly lower open conversion, shorter operative time, and similar median estimated blood loss. Overall, the study demonstrated that it is at least equivalent to standard laparoscopic splenectomy. They enounced that single port splenectomy is an appropriate procedure that can be done safely and may lead to higher patient satisfaction compared to laparoscopic splenectomy. Also, they stated, while articulating instruments and laparoscopes may offer technical advantages, they are not completely necessary for performing single port splenectomy. It is a safe and feasible technique to perform splenectomy for small spleens, but in giant and massive spleens, the reduce port laparoscopic surgery technique is a better choice.

 Laparoscopic splenectomy has become the gold standard for normal size spleens. Massive splenomegaly is not a contraindication for laparoscopic surgery moreover with the develop of the hand assisted technique that is feasible and safe. Morbidity and mortality are equivalent when coparing with regular laparoscopic splenectomy.

## CONCLUSION

The use of reduce port minimizes abdominal trauma and has the hypothetical advantages of shorter postoperative stay, greater pain control, and better cosmesis. Laparoscopic splenectomy for giant cysts by using reduce port trocars is safe and feasible and less invasive.
